# Standards and figure-of-merits for quantifying the performance of triboelectric nanogenerators

**DOI:** 10.1038/ncomms9376

**Published:** 2015-09-25

**Authors:** Yunlong Zi, Simiao Niu, Jie Wang, Zhen Wen, Wei Tang, Zhong Lin Wang

**Affiliations:** 1 School of Materials Science and Engineering, Georgia Institute of Technology, Atlanta, Georgia 30332, USA; 2 Beijing Institute of Nanoenergy and Nanosystems, Chinese Academy of Sciences, Beijing 100083, China

## Abstract

Triboelectric nanogenerators have been invented as a highly efficient, cost-effective and easy scalable energy-harvesting technology for converting ambient mechanical energy into electricity. Four basic working modes have been demonstrated, each of which has different designs to accommodate the corresponding mechanical triggering conditions. A common standard is thus required to quantify the performance of the triboelectric nanogenerators so that their outputs can be compared and evaluated. Here we report figure-of-merits for defining the performance of a triboelectric nanogenerator, which is composed of a structural figure-of-merit related to the structure and a material figure of merit that is the square of the surface charge density. The structural figure-of-merit is derived and simulated to compare the triboelectric nanogenerators with different configurations. A standard method is introduced to quantify the material figure-of-merit for a general surface. This study is likely to establish the standards for developing TENGs towards practical applications and industrialization.

## Abstract (web summary)

Triboelectric nanogenerators are a technology for converting mechanical energy into electricity, but standards for quantifying their performance is lacking. Here, the authors present figure-of-merits to quantitatively evaluate the performance of triboelectric nanogenerators and establish a foundation for further development.

## Graphical abstract

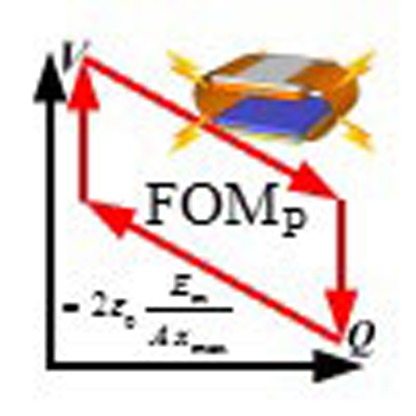

With the rapid growth of portable and wearable electronics, more attentions have been focused on the development of mobile power sources. The current approach for powering these electronic devices is to use batteries, which usually have the limited lifetime and inevitable environmental issues caused by the disposed batteries^1^. Managing, replacing and monitoring the power levels of the batteries that are vastly and dispersedly distributed in almost every corner becomes a huge and impossible task, since it is estimated that the world will have trillions of connection nodes and sensor units by 2020. Alternatively, instead of using batteries, nanogenerators^2^ have been developed to harvest energy from the working environment to make the devices self-powered, including triboelectric nanogenerators (TENG)^1^,^3^,^4^,^5^, piezoelectric nanogenerators^6^,^7^,^8^ and pyroelectric nanogenerators^9^. TENG, which is based on the coupling of triboelectrification and electrostatic induction, has shown advantages such as high output, high energy-conversion efficiency, abundant choices of materials, scalability and flexibility. To improve the performance and broaden the applications of TENG, numerous efforts have been made with focus on both enhancement of the surface charge density *σ* (refs 10, 11) and development of new structures/modes^12^,^13^,^14^,^15^,^16^. However, without a common standard it becomes difficult to evaluate the performance of a TENG. In comparison with its counterparts, standards have been established for alternative power generators, such as Carnot efficiency for heat engines^17^,^18^ and pyroelectric nanogenerators^19^,^20^, figure of merit (ZT) for thermoelectric materials^21^,^22^ and energy-conversion efficiency for solar cells^23^,^24^.

Currently, four basic modes of TENG have been developed^1^,^4^, including vertical contact-separation (CS) mode^3^,^10^,^25^, lateral sliding (LS) mode^12^,^13^,^26^, single-electrode (SE) mode^15^,^27^ and freestanding triboelectric-layer (FT) mode^14^,^16^,^28^. Each mode has its own structure and choice of materials as well as specific mechanical triggering configurations. Taking the CS mode as an example, it is triggered by a vertical periodic driving force that causes a repeated CS of two dissimilar materials that have coated electrodes on the top and bottom surfaces, respectively. As for the LS mode, it is triggered by a LS motion between two dissimilar materials in parallel. To evaluate and compare the performance of the TENGs in different structures/modes, a universal standard has to be introduced to quantify the performance of the TENG, regardless its operation mode.

Here we propose a standard method to quantitatively evaluate TENG's performance from both structure's and the materials' points of view. Starting from the plot of built-up voltage *V*—total transferred charges *Q*, we first propose the TENG operation cycle with maximized energy output. On the basis of this cycle, we propose a performance figure-of-merit (FOMp) for TENG, which consists of a structural FOM (FOM_S_) related to the design of the TENG and a material FOM (FOM_M_) as the square of the surface charge density. To characterize and compare different structures of TENGs, the structural FOM for each configuration of TENG is derived and simulated. A standard method is proposed to quantify the material FOM. The proposed standards will set the foundation for the further applications and industrialization of the TENGs.

## Results

### Cycles for energy output of TENG

**Figure 1:**
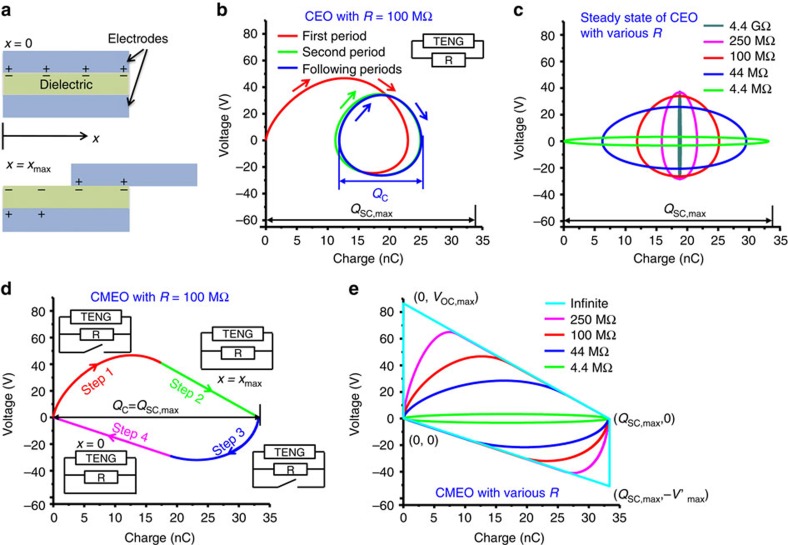
Operation cycles of TENG. (**a**) Schematic diagram of the LS-mode TENG with displacement *x*=0 and *x*=*x*_max_. (**b**) The CEO with load resistance *R*=100 MΩ. The total cycling charge *Q*_C_ was marked and the inset shows the operation circuit. (**c**) The steady-state of CEO with various load resistances. (**d**) The CMEO with load resistance *R*=100 MΩ, with the maximum total cycling charge *Q*_C_=*Q*_SC,max_. The insets show the corresponding status of the switch in circuits during different steps. (**e**) The CMEO with various load resistances. The vertices of the CMEO with infinite load resistances are marked.

The fundamental working principle of the TENG is a conjugation of triboelectrification and electrostatic induction. For a basic TENG, there is at least one pair of triboelectric layers (two pairs for FT mode and one pair for all other modes) that are facing each other for creating opposite triboelectric charges via physical contacts. Besides the triboelectric layers, there are two electrodes (for SE mode, the ground is considered as the second/reference electrode^27^) that are carefully insulated from each other, which are required for the free electrons to be transferred between them through an external load. Triggered by the external mechanical force, there is a periodical relative motion between the triboelectric layers that breaks the balanced distribution of electrostatic charges; as a result, free electrons are driven to flow between the electrodes to build a new equilibrium. Therefore, the governing equations of TENG can be developed on the basis of the relationship among the transferred charges between the electrodes *Q*, the built-up voltage *V* and the relative displacement *x* between the triboelectric layers. The detailed definitions of *x* for different structures of TENG are illustrated in Supplementary Note 1 and Supplementary Fig. 1. We use the most commonly utilized minimum achievable charge reference state (MACRS)^28^; therefore, both the absolute short-circuit transferred charges *Q*_SC_(*x*) and the absolute open-circuit voltage *V*_OC_(*x*) at *x*=0 position are set to be 0. The definitions of the displacement *x* and the two electrodes for an LS-mode TENG are illustrated in Fig. 1a. The maxima of *Q*_SC,max_ and *V*_OC,max_ are expected to be reached at *x=x*_max_ for these basic-mode TENGs.

For a continuous periodic mechanical motion, the electrical output signal from the TENG is also periodically time-dependent. In such a case, the average output power 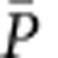, which is related to the load resistance, is used to determine the merits of the TENG. Given a certain period of time *T*, the output energy per cycle *E* can be derived as:


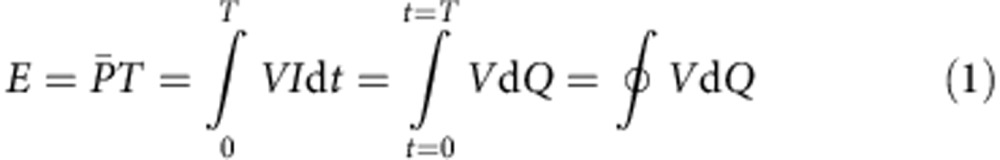


Therefore, the electrostatic states and the energy output of TENG can be represented by the plot of built-up voltage *V* against the transferred charges *Q*. Here we first simulated the *V*–*Q* plot for an LS-mode TENG by finite element method (FEM) operated under external load resistance of 100 MΩ, starting from (*Q*, *V*)=(0, 0). The parameters of this LS-mode TENG was shown in Table 1. From the *V*–*Q* plot, we noticed that the operation of the TENG will go to its steady state after only a few periods (Fig. 1b), and thus we can directly focus on the output of the steady-state operation. Because the steady-state output signal of TENG is periodic in responding to the mechanical triggering, the *V*–*Q* plot should be a closed loop. As indicated by equation (1), the output energy per cycle *E* can be calculated as the encircled area of the closed loop in the *V–Q* curve. The steady-state *V*–*Q* plots for this LS-mode TENG were also simulated by FEM under various external loads, as shown in Fig. 1c. From the encircled areas of these *V–Q* curves, we noticed that the output energy per cycle *E* can be optimized by applying a matched load resistance^26^. The cycles given here can be named as ‘cycles for energy output' (CEO). For each CEO, the difference between the maximum and the minimum transferred charges in its steady state is defined as the total cycling charge *Q*_C_, as marked in Fig. 1b.

### Cycles for maximized energy output of TENG

From Fig. 1b,c, we noticed that for each CEO, the total cycling charge *Q*_C_ was always less than the maximum transferred charges *Q*_SC,max_, especially for cycles with large external load resistances. If we could maximize the *Q*_C_ to be *Q*_SC,max_ for these cycles, the output energy per cycle *E* would be further enhanced. Having noticed that the *Q*_C_=*Q*_SC,max_ occurs at short-circuit condition, we designed following repeated steps to achieve instantaneous short-circuit conditions during operations, with the use of a switch in parallel with the external load (as shown in Fig. 1d): step 1, triboelectric layers displace relatively from *x*=0 to *x*=*x*_max_ at switch off; step 2, turn the switch on to enable *Q*=*Q*_SC,max_ and then turn the switch off; step 3, triboelectric layers displace relatively from *x*=*x*_max_ to *x*=0 at switch off; step 4, turn the switch on to enable *Q*=0, and then turn the switch off. Therefore, the maximized total cycling charge *Q*_C_*=Q*_SC,max_ was enabled by the instantaneous short-circuit conditions in steps 2 and 4, as controlled by the switch. The simulation results with respect to various external load resistances were plotted as Fig. 1e. These cycles can be named as ‘cycles for maximized energy output' (CMEO). Clearly, benefited from the maximized total cycling charges, the output energy per cycle of the CMEO was always higher than that of the CEO with the same load resistance *R*, as observed in the encircled areas in Fig. 1c,e.

We noticed that for the CMEO, higher output energy per cycle can be achieved with larger external load resistance *R* (Fig. 1e). Therefore, the maximized output energy per cycle can be achieved at *R*=+∞, which is the open-circuit condition. We simulated this maximized output energy by simply removing the external load and operating the remaining part of the circuit the same as that for the steps in the CMEO case as stated above. The corresponding *V–Q* curve was plotted as the CMEO with infinite load resistance as shown in Fig. 1e. This CMEO has a trapezoid shape, the vertices of which are determined by the maximum short-circuit transferred charge *Q*_SC,max_, the maximum open-circuit voltage *V*_OC,max_ and the maximum achievable absolute voltage *V*′_max_ at *Q*=*Q*_SC,max_. It can be easily proven that the *V–Q* plots for all kinds of the TENG operations are limited inside the four edges of this trapezoid (Supplementary Note 2). Thus, with the largest encircled area, this cycle has the largest possible output energy per cycle, which can be calculated using the following equation:


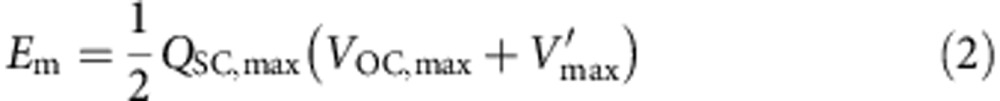


This *E*_m_ can also be derived theoretically as the largest possible output energy per cycle in any operations of the TENG, as shown in the Supplementary Note 3.

### Experimental demonstration of cycles of TENG

**Figure 2:**
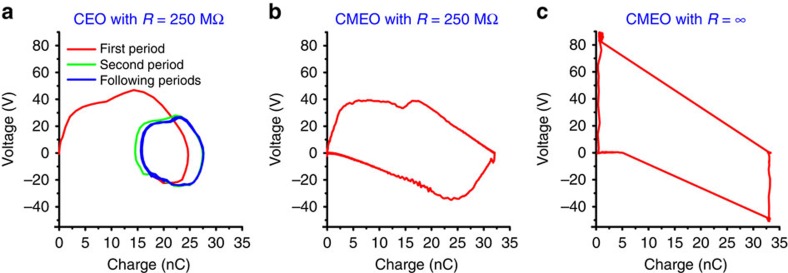
Experimental results of the CMEOs and the CEO. (**a**) The *V–Q* plot of the CEO with external load resistance of 250 MΩ. (**b**) The *V–Q* plot of the CMEO with external load resistance of 250 MΩ. (**c**) The *V–Q* plot of the CMEO with infinite external load resistance.

Here we also demonstrated that the CEO and the CMEO can be easily achieved experimentally. An LS-mode TENG with parameters close to that in Table 1 was fabricated. An aluminium (Al) foil was used as one electrode and the motion part. The copper (Cu) layer deposited on the fluorinated ethylene propylene (FEP) film was utilized as the other electrode. The Al foil and the FEP film are the pair of triboelectric layers. First, the *V*–*Q* plot of CEO was demonstrated by using an external load resistance of 250 MΩ, as shown in Fig. 2a. We noticed that the steady state of the CEO was achieved only after a few periods of operation. And then, controlled by a switch in series, the *V*–*Q* plot of CMEO with external load resistances of 250 MΩ and infinite was also demonstrated, as shown in Fig. 2b,c, respectively. All of the features of the three *V*–*Q* plots are very consistent with the simulated results as shown in Fig. 1. We also measured and plotted the *V*–*Q* plots of CEO and CMEO with various external load resistances as Supplementary Fig. 3. We found that the trends of the characteristics with the varied resistances of these plots are very consistent with that of the simulated results. We can easily conclude from the encircled areas that the output energy per cycle of the CMEO with infinite external load resistance was the highest one in that of these cycles. Each output energy per cycle was calculated using equation (1), as shown in Table 2. These results demonstrated and further confirmed that the experimentally achievable CMEO can harvest the maximized output energy per cycle. In fact, the instantaneous discharging TENG with a switch triggered by the motion of TENG, which was actually operated in CMEO, has been demonstrated to have gigantic enhanced instantaneous power compared with the traditional TENG operated in CEO^29^.

### Figure-of-merits of TENG

For the TENG operating in CMEO with infinite load resistance, the period *T* includes two parts of time. One part is from the relative motion in TENG, and the other part is from the discharging process in short-circuit condition. With the minimal resistance in short-circuit condition, the second part of *T* can be short enough so that it can be omitted (less than 0.4 ms experimentally as reported^29^). Thus, the average output power 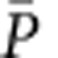 at CMEO should satisfy:


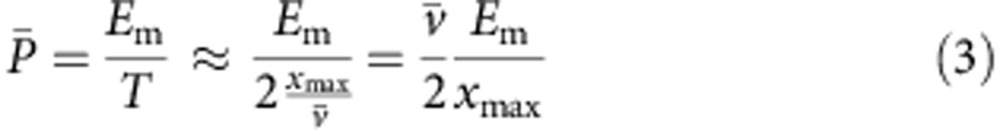


where 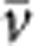 is the average velocity value of the relative motion in TENG, which depends on the input mechanical motions. In this equation, *E*_m_/*x*_max_ is the only term that depends on the characteristics of the TENG itself.

On the other hand, the energy-conversion efficiency of the TENG can be expressed as (at CMEO with *R*=+∞):


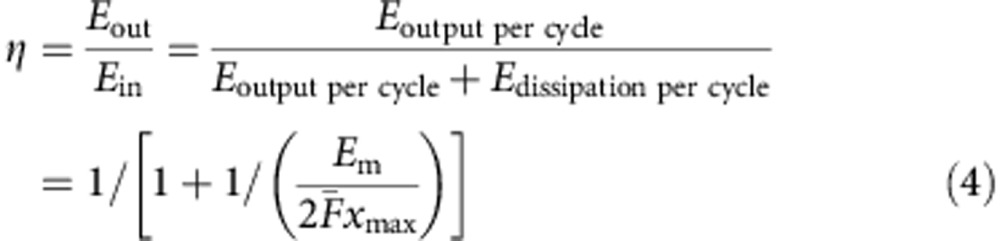


Here 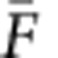 stands for the average dissipative force during the operation of the TENG. This force can be frictional force, air resistance force or others.

Therefore, from both equations (3) and (4) we conclude that the term *E*_m_/*x*_max_ determines both the average power and the energy-conversion efficiency from the characteristics of TENG itself. We also notice that as indicated in equation (2), *E*_m_ contains *Q*_SC,max_ that is proportional to the triboelectrification area *A*. Therefore, to exclude the effect of the TENG size on the output energy, the area *A* should be placed in denominator of this term. Owing to the reasons stated above, we confirm that the term *E*_m_/*Ax*_max_ determines the merits of TENG.

We notice that in equation (2), *Q*_SC,max_, *V*_OC,max_ and *V*′_max_ are all proportional to the surface charge density *σ*. Therefore, *E*_m_ is proportional to the square of the surface charge density *σ*. Then, we can define a dimensionless structural FOM (FOM_S_) of TENG, as the factor only depends on the structural parameters and *x*_max_:


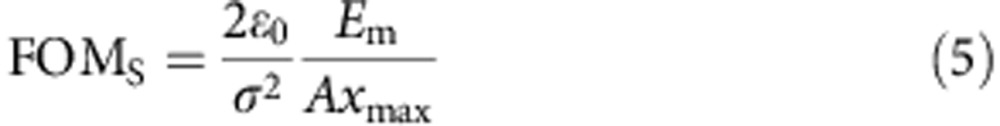


Here *ɛ*_0_ is the permittivity of the vacuum. This structural FOM represents the merit of the TENG from the structural design. And then the performance FOM (FOM_P_) of TENG can be defined as:


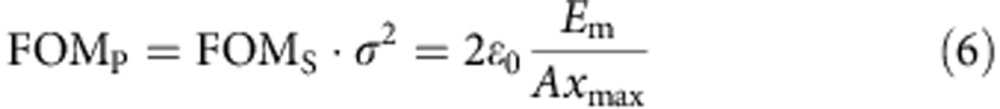


in which the *σ*^2^ can be the FOM_M_, which is the only component related to the material properties. The FOM_P_ can be considered as the universal standard to evaluate varieties of TENGs, since it is directly proportional to the greatest possible average output power and related to the highest achievable energy-conversion efficiency, regardless of the mode and the size of the TENG.

### The structural FOM of different modes of TENG

**Figure 3:**
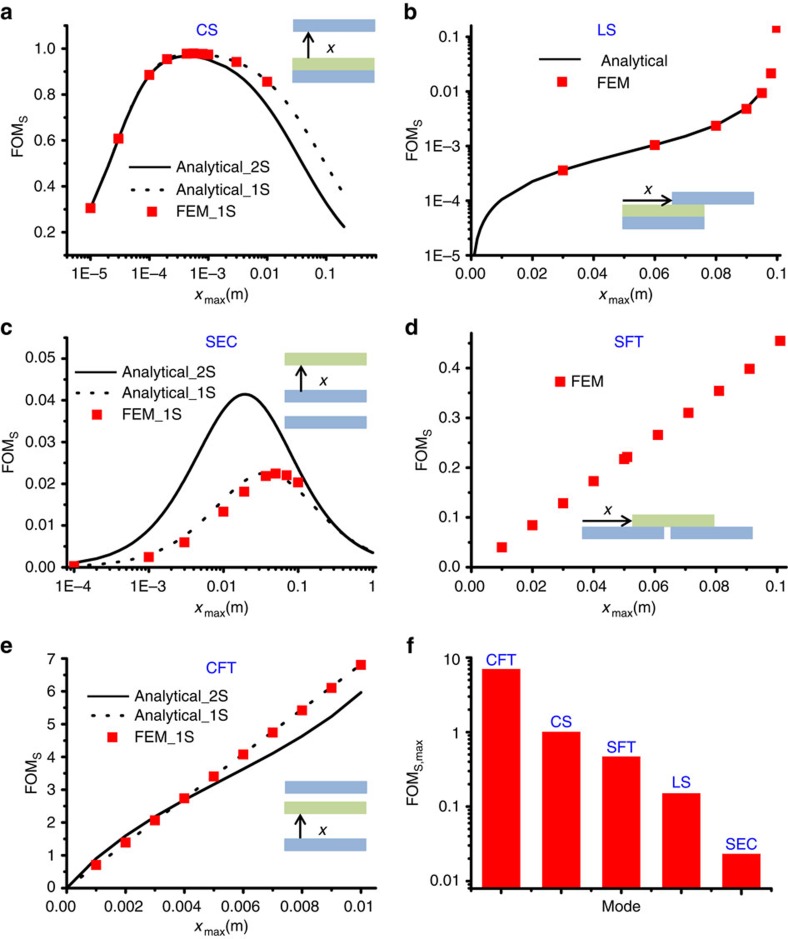
FOM_S_ versus *x*_max_ for different TENG structures. (**a**–**e**) The FOM_S_ for vertical CS mode, LS mode, single-electrode contact (SEC) structure, sliding SFT structure and contact freestanding triboelectric-layer (CFT) structure calculated by analytical formulas and FEM simulation. The insets show the corresponding schematic diagrams of the corresponding structures. 1S and 2S stand for calculations and simulations considering 1-side and 2-side side effects of the non-ideal parallel-plate capacitor. (**f**) The maximum structural FOM (FOM_S,max_) of different structures extracted from FEM simulations.

Currently, four basic modes of TENGs have been developed for various applications^1^,^4^. To compare these TENGs from the structural design's point of view, the structural FOM of CS mode, LS mode, SE contact (SEC) structure in SE mode, sliding FT (SFT) and contact FT (CFT) structures in FT mode TENGs, were calculated by their analytical formulae and simulated by FEM, with the same surface charge density *σ* and area *A*. The detailed parameters and analytical formulae were described in Supplementary Notes 4 and 5, Supplementary Table 1 and Supplementary Fig. 1. The calculated structural FOMs for the five structures with respect to varied maximum displacement *x*_max_ were plotted and compared in Fig. 3. For CS, SEC and CFT structures, the non-ideal parallel-plate capacitances considering both one-side and two-side edge effects were used for calculations by analytical formulae^30^, while in the FEM simulation only one-side edge effect was considered. The calculated results from the analytical formulae fit the FEM simulation results very well. We can extract the maximum value of the structural FOM (FOM_S,max_) from FEM simulations as a standard criterion for evaluating the structures. As shown in Fig. 3f and Table 3, we found for FOM_S,max_:


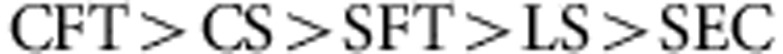


Here we can make several general conclusions. First, the performance of the paired-electrode TENGs is dramatically better than that of SE TENGs with the same size and materials because of the limited transferred charges and suppressed built-in voltages in SE TENG, which has been reported previously^27^. Second, the performance of TENG triggered by CS action is better than that of TENG triggered by sliding (assuming that the triboelectric charge densities created by CS and sliding are the same) because, to achieve the same *V*_OC,max_, a much higher *x*_max_ of TENG triggered by sliding is required compared with that of TENG triggered by CS, resulting in a smaller FOM_S,max_. Finally, the freestanding configuration enhances the performance by dramatically decreasing the capacitance between electrodes. The high FOM_S,max_ of CFT structure is also benefited by the large transferred charges induced by the double-side triboelectrification of the middle dielectric layer during operation (as shown in Supplementary Fig. 1e).

### The measurement of *σ* and the dimensionless FOM_M_

The triboelectric surface charge density *σ* is the only material-related parameter in FOM_P_ as shown in equation (6). The optimization of *σ* would enhance the performance of TENG significantly since the FOM_P_ is proportional to *σ*^2^. This surface charge density was determined by the triboelectric performance of the materials in contact. Since the existing triboelectric series is a qualitative measure of the materials' triboelectric performances^4^, a quantitative measuring matrix is required for TENGs.

Current studies of the triboelectric performance are influenced by the low contact intimacy induced by the nanometre-to-micrometre-level surface roughness in solid materials^31^,^32^,^33^. Therefore, the measured surface charge density by solid-solid contact was not able to reach its highest possible value. To overcome this limitation of the solid surface and to build the measuring matrix for triboelectric performance of materials, we can utilize liquid metals such as liquid gallium (Ga, melting point: 29.8 °C), galinstan (a eutectic alloy consisting of 68.5 wt% gallium, 21.5 wt% indium and 10 wt% tin, melting point: −19 °C) and mercury (melting point: −38.8 °C) as one triboelectrification material. It is expected that, by using these liquid metals, the contact intimacy could be greatly enhanced since the liquid metals are shape-adaptive to the solid surfaces^34^,^35^. Here the non-toxic liquid gallium and galinstan were utilized in our experiments. To prevent them from being oxidized that would happen very fast for all the liquid metals containing gallium^34^,^35^,^36^,^37^, the experiments were performed in a glove box with argon environment and the fixed condition of room temperature, 1 atm and 0.006% in relative humidity (RH).

**Figure 4:**
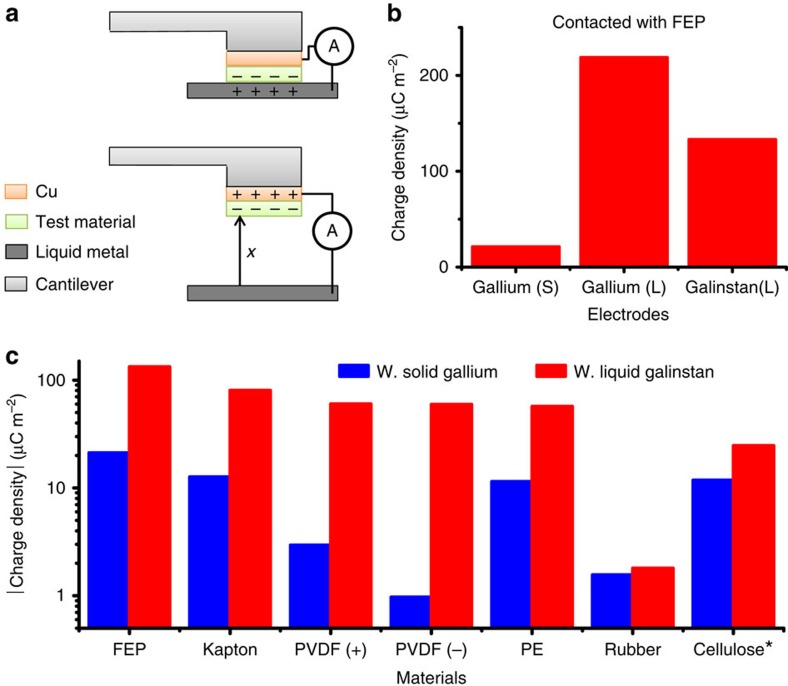
The standard *σ* measured by liquid metal as one electrode. (**a**) The measurement set-up. (**b**) The charge density measured by contacting FEP with solid gallium, liquid gallium and galinstan. (**c**) The absolute charge density measured by contacting different materials with solid gallium and liquid galinstan. Among them, cellulose was measured to be more positive than galinstan (as marked by *).

The set-up of the measurement experiment is shown in Fig. 4a. First, the tested material was deposited with Cu thin film as one electrode, and the liquid galinstan (from rotometals) or liquid gallium (from VWR) was used as the other electrode. When the material surface was contacted with the liquid metal surface, the charges were created on both the material and liquid metal surfaces by triboelectrification effect. And then, after the material along with the Cu electrode was lifted above the liquid metal surface for a height much larger (over 100 times) than the thickness of the materials, the charges induced by triboelectrification would be approximately fully transferred to the Cu electrode in short-circuit condition. The transferred charges were measured as the triboelectric charges, and then the charge density was calculated by the charges over the area of the triboelectrification surface. To make comparisons, solid gallium was also utilized to replace liquid metals as one electrode for charge density measurements. The good repeatability of charge transfer was observed during multiple CS processes (Supplementary Fig. 2). The tested materials included FEP, Kapton, polarized polyvinylidene fluoride (PVDF), polyethylene (PE), nature rubber and cellulose. (See discussions in Supplementary Note 6.) All of the measurement results are shown in Fig. 4b,c. When the tested material is more negative than the liquid metal, the measured charge density is recorded as a positive value; when the material is more positive than liquid metal, the measured charge density is recorded as a negative value (such as cellulose).

As we noticed from the results, the measured charge densities by contacting tested materials with a liquid metal are always larger than that by contacting the material with the corresponding solid metal. This is because with liquid metals used as one contact, the contact intimacy can be greatly enhanced. In fact, by using liquid mercury as the contact, even higher surface charge densities can be achieved^33^. The varied charge densities measured by contacting the same material with different liquid metals might be because of the different capabilities of the liquid metals to absorb the electrons. The triboelectric order as derived from our measurement is: (most negative) FEP—Kapton—PVDF—PE—nature rubber—Galinstan—Cellulose (most positive), which are consistent with existing triboelectric series as reported^4^. Triboelectric performance of each material can be standardly quantified using the triboelectric surface charge density with respect to a certain liquid metal. For example, the triboelectric performance of FEP can be quantified as *σ*_FEP/galinstan_=133.24 μC m^−2^ with respect to galinstan, or *σ*_FEP/Ga(L)_=218.64 μC m^−2^ with respect to liquid gallium. FEP (Teflon) is usually considered as the most triboelectric negative material (see discussions in Supplementary Note 7); galinstan should be about in the middle position of the triboelectric series since it is close to nature rubber and it is more negative than cellulose. Therefore, we can consider the surface charge densities while contacting FEP with galinstan as the reference triboelectric charge densities. Then, the normalized triboelectric charge density *σ*_N_ and dimensionless material FOM (FOM_DM_) for triboelectrification (with respect to the charge density of FEP contacting with galinstan) can be defined as:


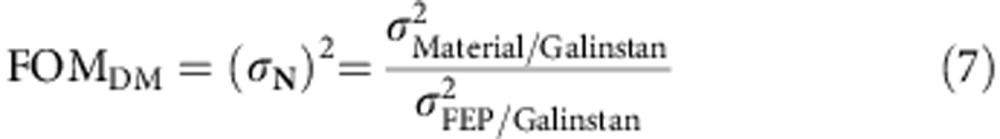


Then the normalized triboelectric charge densities and FOM_DM_ of measured materials are listed in Table 4. For a certain material, if *σ*_N_<0, then the material is more positive than the reference liquid metal; if 0<*σ*_N_<1, then this material is more negative than the reference liquid metal and more positive than FEP; if *σ*_N_>1, then this material is more negative than FEP.

## Discussion

We have developed methods for standardized evaluations on the performance of TENGs. Starting from the built-up voltage *V*-transferred charge *Q* plot, the CMEO with infinite load resistance was derived to have the maximized output energy per cycle, which represents the maximum energy production of TENG, similar to the Carnot cycle in heat engines. On the basis of the maximum output energy per cycle, and considering both the maximized energy-conversion efficiency and the maximized average output power, the FOM_P_ was derived to evaluate each TENG design, composed by a structural FOM and a FOM_M_. The structural FOMs for different structures of TENGs were simulated by analytical formulae and FEM, respectively, showing the maximum value of structural FOM for each TENG structure. The standard evaluation of the FOM_M_ was also demonstrated by measuring triboelectric surface charge density via contacting the materials with liquid metals, and then the normalized triboelectric charge density and FOM_DM_ were defined and derived for various materials. The standards and evaluation methods provide here set the foundation for the further applications and industrialization of TENG technology.

## Methods

### Fabrication and operations of the LS-mode TENG

The static part of the TENG was fabricated by depositing copper (Cu) film as electrode on a 50-μm-thick FEP film, and then the Cu/FEP film was attached on an acrylic board with the Cu side facing to the acrylic board. The aluminium (Al) foil attached on another acrylic board was used as the motion part. To operate the TENG, the motion part was mounted on a linear motor and the static part was mounted on a three-dimensional stage, and the FEP surface and the Al foil were placed to face to each other. The linear motor was controlled to move periodically with displacement of 3.5 cm. For the CEO, the linear motor was controlled to move continuously with a period of ∼0.34 s, with an external resistor with resistance of 250 MΩ used. For the CMEO, after each sliding motion in one direction, the motor was hold with a wait time of 5 s, and the switch was triggered right at the beginning and the end of the 5-s wait time period. The resistor was either used or not used to make the external load resistance of 250 MΩ and infinite. The voltage and transferred charge were measured using a Keithley 6514 system electrometer.

### FEM simulation methods

The simulation process includes electrostatic simulation and load-circuit simulation. For electrostatic simulation, the COMSOL electrostatic module is utilized to calculate *V*_OC_(*x*), *C*(*x*) and *Q*_SC_(*x*) under MACRS; therefore, all the information required for the largest possible energy output per cycle *E*_m_ can be obtained and the structural FOM can be calculated.

If a TENG is connected with a load resistance, the governing equation combining the resistive load and TENG is shown as:


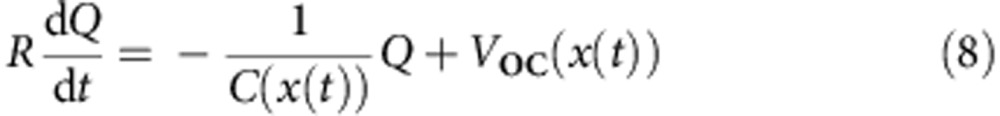


The voltage output is calculated as:


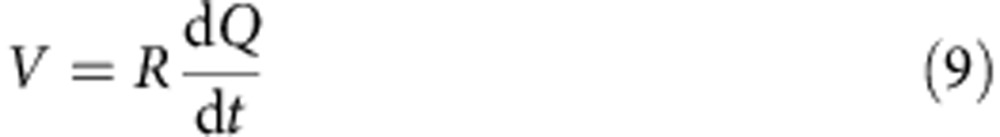


As an example of typical mechanical motion, we utilize the harmonic mechanical motion in which the *x*(*t*) is defined as


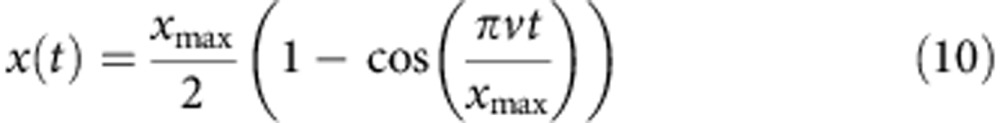


To calculate the steady-state output for CEO, we can simply apply the periodic boundary condition of *Q* (*t*=0)=*Q* (*t*=*T*), where *T* is the period of external periodic mechanical motion.

To calculate the output for CMEO, we need to solve the differential equation for each half cycle. At the first half cycle, when the electrostatic potential is balanced, the boundary condition is: (under MACRS) *Q* (*t*=0)=0. At the second half cycle, when the electrostatic potential is balanced again, the boundary condition is *Q* (*t*=*T*/2)=*Q*_SC,max_.

### Measurement of surface charge density with liquid metals

The cantilever was made by an acrylic block (with area of 19 × 19 mm) attached to an acrylic board. The tested material was cut to be 19 × 19 mm and was deposited with copper (Cu) film as the electrode. This tested material was attached on the acrylic block in the cantilever with the Cu electrode facing to the surface of the block. This whole cantilever was mounted on a three-dimensional stage, and the cantilever can be lifted and pushed down manually. And then this structure along with a plastic Petri dish and the liquid metals was moved to a glove box with argon environment. The environmental condition was fixed at room temperature, 1 atm and 0.006% RH. For liquid gallium, the unpacked bottle was heated on the hot plated in 50 °C for 5 min before moving into the glove box. In the glove box, the liquid metal was poured to the Petri dish, which was placed right below the tested material. Two copper wires were connected on the Cu film and the liquid metal as two electrodes, respectively. The lowest position of the cantilever was carefully adjusted to make sure precisely right contact between the tested material and the liquid metal; therefore, there is actually very minimum pressure applied on the liquid metal while contacting. And then the cantilever was lifted and pushed down periodically by hands to achieve CS process, with the period of ∼4–12 s. The transferred charge was measured using a Keithley 6514 system electrometer. The charge density measurement with the solid gallium was performed after the solidification of liquid gallium.

## 

## Additional information

**How to cite this article:** Zi, Y. *et al.* Standards and figure-of-merits for quantifying the performance of triboelectric nanogenerators. *Nat. Commun.* 6:8376 doi: 10.1038/ncomms9376 (2015).

## Supplementary Material

Supplementary Information Supplementary Figures 1-3, Supplementary Table 1, Supplementary Notes 1-7 and Supplementary References

## Figures and Tables

**Table 1:** Parameters used for simulating the LS mode TENG.

Dielectric effective thickness, *d*_0_=Σ*d*_i_/*ɛ*_ri_	90.8 μm
Triboelectrification area *A* (length *l* × width *w*)	8.5 × 7.9 cm
Maximum displacement, *x*_max_	3.5 cm
Surface charge density, *σ*	12 μC m^−2^

LS, lateral sliding; TENG, triboelectric nanogenerator.

**Table 2:** Output energy per cycle for the three cycles operated in the LS-mode TENG.

**Cycle type**	**Output energy per cycle (μJ)**
CMEO with *R*=+∞	1.99
CMEO with *R*=250 MΩ	1.48
CEO with *R*=250 MΩ	0.47

CMEO, cycles for maximized energy output; LS, lateral sliding; TENG, triboelectric nanogenerator.

**Table 3:** The simulated FOM_S,max_.

**Structure**	**FOM**_**S,max**_
CFT	6.81
CS	0.98
SFT	0.45
LS	0.15
SEC	0.022

CFT, contact FT; CS, contact separation; FOM, figure-of-merit; FOM_S,max_, maximum value of the structural FOM; FT, freestanding triboelectric layer; LS, lateral sliding; SEC, single-electrode contact; SFT, sliding FT.

**Table 4:** *σ*_N_ and FOM_DM_ of different materials.

**Materials**	**Normalized triboelectric charge density**	**Dimensionless material figure-of-merit**	**Position in triboelectric series**
FEP	1	1	Most, −
Kapton	0.60	0.36	
PVDF	0.45	0.20	
PE	0.43	0.18	
Nature rubber	0.0135	0.000183	
Galinstan	0	0	About middle
Cellulose	−0.185	0.0342	+

FEP, fluorinated ethylene propylene; FOM_DM_, dimensionless material figure-of-merit; PE, polyethylene; PVDF, polyvinylidene fluoride.

These results are with respect to the charge density of contacting FEP with galinstan.
