# Supplementary material for: Standards and figure-of-merits for quantifying the performance of triboelectric nanogenerators
Source: Nat Commun. 2015 Sep 25;6:8376. doi: 10.1038/ncomms9376 (PMC4598564; doi:10.1038/ncomms9376)
Supplement: Supplementary Information — Supplementary Figures 1-3, Supplementary Table 1, Supplementary Notes 1-7 and Supplementary References [file ncomms9376-s1.pdf]

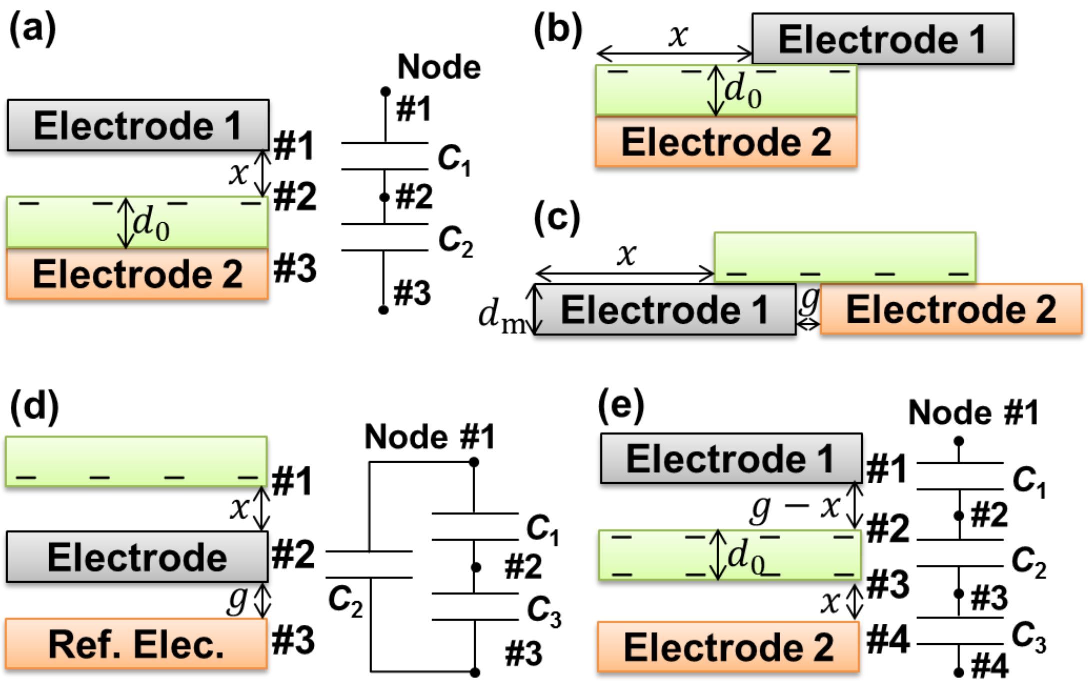

Supplementary Figure 1. Illustration of symbols in 5 structures of TENG. (a) Vertical contact-separation (CS) mode TENG with capacitance $C_1(x)$ between electrode 1 (node #1) and triboelectric surface of the dielectric layer (node #2), and capacitance C_2 between node #2 and electrode 2 (node #3); (b) Lateral sliding (LS) mode TENG; (c) Sliding freestanding triboelectric-layer (SFT) structure TENG; (d) Single-electrode contact (SEC) structure TENG with capacitance $C_1(x)$ between triboelectric surface of the dielectric layer (node #1) and the electrode (node #2), capacitance $C_2(x)$ between node #1 and the reference electrode (node #3), and capacitance $C_3(x)$ between nodes #2 and #3. These capacitances are "virtual" capacitances as described in Ref. [1]; (e) Contact freestanding triboelectric-layer (CFT) structure TENG with capacitance $C_1(x)$ between electrode 1 (node #1) and the top triboelectric surface of the dielectric layer (node #2), capacitance C_2 between node #2 and the bottom triboelectric surface of the dielectric layer (node #3), and capacitance $C_3(x)$ between nodes #3 and electrode 2 (node #4).

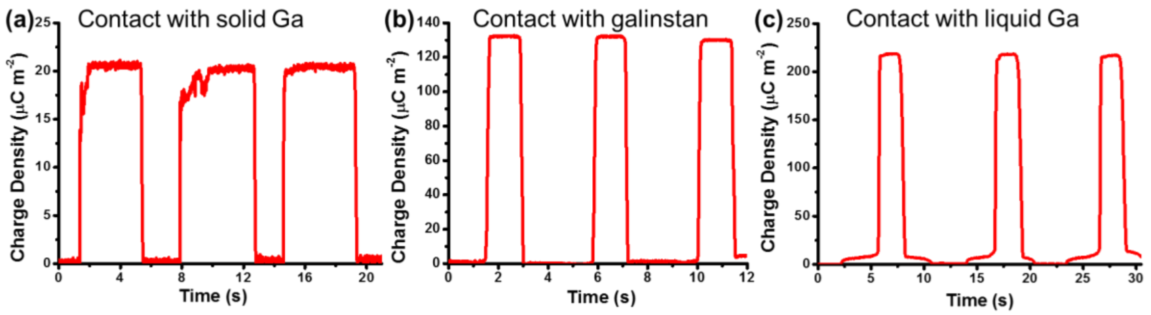

Supplementary Figure 2. Surface charge density measurement by contacting FEP with (a) solid gallium, (b) liquid galinstan, and (c) liquid gallium.

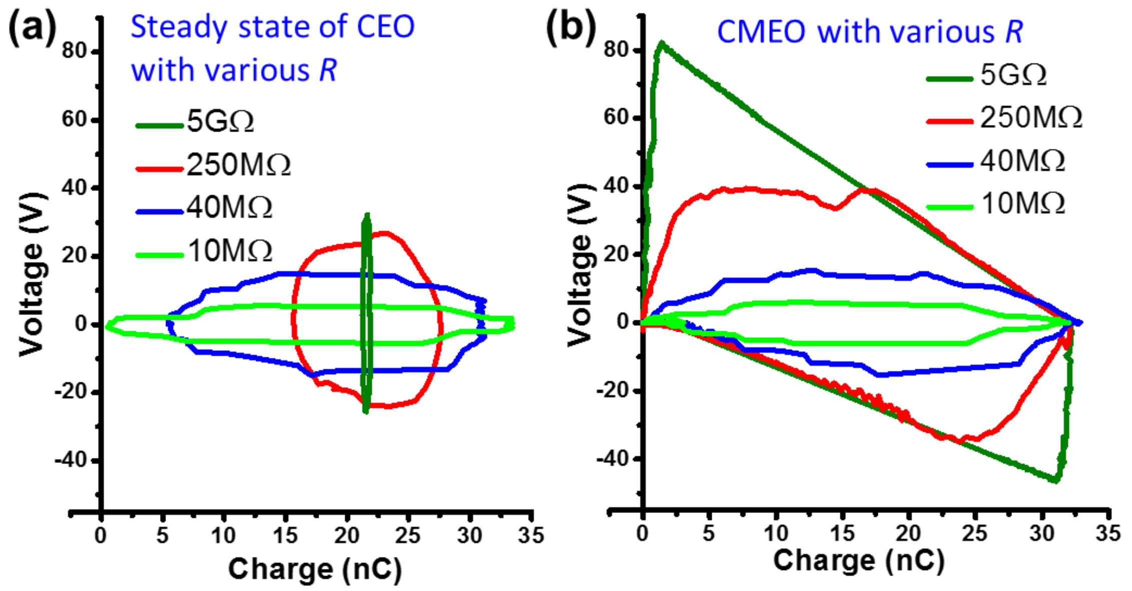

Supplementary Figure 3. The experimental measured (a) steady state of CEO and (b) CMEO with various external load resistances.

Supplementary Table 1. Parameters used in simulation for different structures of TENG

Structure		CS	LS	SEC	SFT	CFT
Dielectric		Effective thickness $d_0 = \Sigma d_i/\varepsilon_{ri} = 50$ μm				
Air		Relative dielectric constant $\varepsilon_r = 1$				
Electr-odes	Size	Area A = Length $l \times$ Width w = 0.1 m × 0.1 m = 0.01 m^2				
	Thickness	d_m = 1 μm				
Triboelectric surface charge density		σ = 10 μC m^{-2}				
Displacement x_{max}		0~0.2 m	0~0.1 m	0~1 m	0~0.101 m	0~0.01 m
(Ref. to Fig. S1) Gap g between electrodes		-	-	1 cm	1 mm	1 cm

Supplementary Note 1: The definitions of displacement *x* in different structures of TENG: Usually, we define the status while one pair of the triboelectric layers are fully contacting with each other as $x = 0$, and $x = x_{max}$ is defined at the farthest achievable displacement. (Please be noticed that actually the x_{max} can be picked at any displacement larger than 0. In application, the x_{max} is fixed inside a range which depends on the structural design.) The detailed illustration for the different structures of TENG was shown in Supplementary Figure 1.

Supplementary Note 2: The four boundary lines of *V*-*Q* plots of TENG: We only consider the charge Q with $0 \le Q \le Q_{SC,max}$ since the motion part only operates between $x = 0$ and $x = x_{max}$. $0 \le Q \le Q_{SC,max}$ represents two of the four boundary lines.

At arbitrary displacement x, the capacitance was fixed at $C(x)$. Therefore, as the electrical potential superposition of the open-circuit voltage and the voltage drop due to the charge transfer, the total voltage was derived as:

$$V = -\frac{Q}{C(x)} + V_{OC}(x) \qquad (1)$$

Here we defined a variable $V'(x)$, which is the absolute voltage value when $Q = Q_{\mathrm{SC,max}}$ at displacement x. So from the definitions, at fixed x, (Q, V) plots (Supplementary Equation (1)) should include points of ($Q_{\mathrm{SC}}(x)$, 0), (0, $V_{\mathrm{OC}}(x)$) and ($Q_{\mathrm{SC,max}}$, -$V'(x)$). By putting these points into Supplementary Equation (1) we can get:

$$\begin{cases} V'(x) = \left| -\frac{Q_{\mathrm{SC,max}}}{C(x)} + V_{\mathrm{OC}}(x) \right| = \frac{Q_{\mathrm{SC,max}}}{C(x)} - V_{\mathrm{OC}}(x) & \text{(2a)} \\ 0 = -\frac{Q_{\mathrm{SC}}(x)}{C(x)} + V_{\mathrm{OC}}(x) & \text{(2b)} \end{cases}$$

Then the relationship among $Q_{\mathrm{SC}}(x)$, $V_{\mathrm{OC}}(x)$, $C(x)$ and $V'(x)$ can be shown as:

$$C(x) = \frac{Q_{\mathrm{SC}}(x)}{V_{\mathrm{OC}}(x)} = \frac{Q_{\mathrm{SC,max}} - Q_{\mathrm{SC}}(x)}{V'(x)} \tag{3}$$

If we replace $C(x)$ in Supplementary Equation (1) by Supplementary Equation (3), we can get:

$$\begin{cases} \frac{V}{V_{\mathrm{OC}}(x)} + \frac{Q}{Q_{\mathrm{SC}}(x)} = 1 & \text{(4a)} \\ -\frac{V}{V'(x)} + \frac{Q_{\mathrm{SC.max}} - Q}{Q_{\mathrm{SC.max}} - Q_{\mathrm{SC}}(x)} = 1 & \text{(4b)} \end{cases}$$

As we indicated in note (31) in the article, $V_{\mathrm{OC}}(x)$ and $Q_{\mathrm{SC}}(x)$ always increase with the increase of x. Therefore, at $x = 0$, $V_{\mathrm{OC}}(x)$ and $Q_{\mathrm{SC}}(x)$ should achieve their minimum value $V_{\mathrm{OC}}(0) = 0$ and $Q_{\mathrm{SC}}(0) = 0$; and at $x = x_{\mathrm{max}}$, $V_{\mathrm{OC}}(x)$ and $Q_{\mathrm{SC}}(x)$ should achieve their maximum value $V_{\mathrm{OC}}(x_{\mathrm{max}}) = V_{\mathrm{OC,max}}$ and $Q_{\mathrm{SC}}(x_{\mathrm{max}}) = Q_{\mathrm{SC,max}}$. Therefore, $0 \leq Q_{\mathrm{SC}}(x) \leq Q_{\mathrm{SC,max}}$, $0 \leq V_{\mathrm{OC}}(x) \leq V_{\mathrm{OC,max}}$.

We noticed if we define a new displacement $x' = x_{\max} - x$, and redefine the MACRS in this new coordinate system as $V_{OC}'(x') = 0$ and $Q_{SC}'(x') = 0$ at $x' = 0$. There would be $Q_{SC}'(x') = Q_{SC,\max} - Q_{SC}(x)$ and $C'(x') = C(x)$ from the definitions. Therefore,

$$V_{OC}'(x') = \frac{Q_{SC}'(x')}{C'(x')} = \frac{Q_{SC,\max} - Q_{SC}(x)}{C(x)} = V'(x) \tag{5}$$

So as the open-circuit voltage at this new charge reference state, $V_{OC}'(x') = V'(x)$ should achieve the minimum value 0 at $x' = 0$ and the maximum value $V'_{\max}$ at $x' = x_{\max}$. Then there would be $0 \le V'(x) \le V'_{\max}$.

So for arbitrary (Q, V) with displacement x and satisfying Supplementary Equations (4a) and (4b):

When $V \ge 0$, $\frac{V}{V'_{\max}} + \frac{Q}{Q_{SC,\max}} \ge 0$ is always valid, and besides,

$$\frac{V}{V_{OC,\max}} + \frac{Q}{Q_{SC,\max}} \le \frac{V}{V_{OC}(x)} + \frac{Q}{Q_{SC}(x)} = 1;$$

When $V < 0$, $\frac{V}{V_{OC,\max}} + \frac{Q}{Q_{SC,\max}} < \frac{Q}{Q_{SC,\max}} \le 1$ is always valid, and besides,

$$\frac{V}{V'_{\max}} + \frac{Q}{Q_{SC,\max}} = \frac{V}{V'_{\max}} - \frac{Q_{SC,\max} - Q}{Q_{SC,\max}} + 1 \ge \frac{V}{V'(x)} - \frac{Q_{SC,\max} - Q}{Q_{SC,\max} - Q_{SC}(x)} + 1 = 0.$$

Therefore, for any (Q, V) in any states of the TENG, $\begin{cases} \frac{V}{V_{OC,\max}} + \frac{Q}{Q_{SC,\max}} \le 1 \\ \frac{V}{V'_{\max}} + \frac{Q}{Q_{SC,\max}} \ge 0 \end{cases}$, consequently:

$$\begin{cases} V \le V_{OC,\max} - V_{OC,\max} \frac{Q}{Q_{SC,\max}} & \text{(6a)} \\ -V \le V'_{\max} \frac{Q}{Q_{SC,\max}} & \text{(6b)} \end{cases}$$

Supplementary Equations (6a) and (6b) represent the other two of the four boundary lines. Then the four boundary lines of the *V*-*Q* curves were fixed.

Supplementary Note 3: E_{m} as the largest possible output energy per cycle: For TENG under arbitrary operations, energy per cycle $E = \oint V \mathrm{d}Q$, we can divide the route into two parts:

Part 1: when $V \geq 0$, $\mathrm{d}Q/\mathrm{d}t = I = V/R \geq 0$, so Q will always increase at $V \geq 0$ (assume it is from $Q_{1\mathrm{min}}$ to $Q_{1\mathrm{max}}$);

Part 2: when $V < 0$, $\mathrm{d}Q/\mathrm{d}t = I = V/R < 0$, so Q will always decrease at $V < 0$ (assume it is from $Q_{2\mathrm{max}}$ to $Q_{2\mathrm{min}}$);

So: $E = \oint V \mathrm{d}Q$

= (Part 1: $V \geq 0$) $\int_{Q_{1\min}}^{Q_{1\max}} V \mathrm{d}Q$ + (Part 2: $V < 0$) $\int_{Q_{2\max}}^{Q_{2\min}} V \mathrm{d}Q$

= (Part 1: $V \geq 0$) $\int_{Q_{1\min}}^{Q_{1\max}} V \mathrm{d}Q$ + (Part 2: $V < 0$) $\int_{Q_{2\min}}^{Q_{2\max}} \left(-V\right) \mathrm{d}Q$

$\leq$ (Part 1) $\int_{Q_{1\min}}^{Q_{1\max}} \left(V_{\mathrm{OC,max}} - V_{\mathrm{OC,max}} \frac{Q}{Q_{\mathrm{SC,max}}} \right) \mathrm{d}Q$ + (Part 2) $\int_{Q_{2\min}}^{Q_{2\max}} \left(V'_{\mathrm{max}} \frac{Q}{Q_{\mathrm{SC,max}}} \right) \mathrm{d}Q$

due to Supplementary Equations (6a) and (6b)

$\leq \int_{0}^{Q_{\mathrm{SC,max}}} \left(V_{\mathrm{OC,max}} - V_{\mathrm{OC,max}} \frac{Q}{Q_{\mathrm{SC,max}}} + V'_{\mathrm{max}} \frac{Q}{Q_{\mathrm{SC,max}}} \right) \mathrm{d}Q$ due to $0 \leq Q \leq Q_{\mathrm{SC,max}}$

$= \frac{1}{2} Q_{\mathrm{SC,max}} \left(V_{\mathrm{OC,max}} + V'_{\mathrm{max}} \right) = E_{\mathrm{m}}$

Supplementary Note 4: The parameters of TENGs for simulation: We assume that each TENG takes surface charge density σ. All the electrodes have the size of area (A) =

length (l) × width (w). The sliding direction in LS and SFT structures is along the length direction. The maximum displacement of the motion part is $x_{\max}$, and the effective thickness of the dielectric layer is defined as $d_0 = \Sigma_i d_i/\varepsilon_{ri}$ in which d_i and ε_{ri} represent the thickness and dielectric constant of each dielectric layer, respectively. The capacitances between different electrodes and triboelectric surfaces are marked in Supplementary Figure 1, and $C_{\text{Total}}(x)$ is the total capacitance, which is the capacitance between two electrodes. The parameters for each structure of TENG were listed in Supplementary Figure 1 and Supplementary Table 1.

Supplementary Note 5: The analytical formulas for structural FOM: The analytical formulas of $V_{\text{OC,max}}$ and $Q_{\text{SC,max}}$ in CS, LS, SEC and CFT structures have been given by previous papers[1-4]. For these structures, the $V'_{\max}$ can also be derived by the models in these papers. The LS structure with $x_{\max} > 0.95l$ and SFT structure were not calculated due to the lack of analytical formulas. That is because in the both cases, the capacitances C_{Total} are between two fully misaligned parallel plates, which currently don't have appropriate analytical formulas. And besides, the analytical formulas of $Q_{\text{SC}}(x)$ cannot be derived, either, since they are strongly related to the side effects.

The formulas of $V_{\text{OC,max}}$, $Q_{\text{SC,max}}$ and $V'_{\max}$ used were listed below (Please refer to the previous section for the meaning of symbols):

CS:

$$V_{\text{OC,max}} = \frac{\sigma A}{C_1\left(x_{\max}\right)} \tag{7}$$

$$Q_{\text{SC,max}} = V_{\text{OC,max}} C_{\text{Total}} = \sigma A \frac{C_2}{C_1\left(x_{\max}\right) + C_2} \tag{8}$$

$$V'_{\max} = \frac{Q_{\mathrm{SC,max}}}{C_2} = \frac{\sigma A}{C_1(x_{\max}) + C_2} \tag{9}$$

LS (only for $0 \le x_{\max} \le 0.95l$):

$$V_{\mathrm{OC,max}} = \frac{\sigma x_{\max}}{\varepsilon_0 (l - x_{\max})} d_0 \tag{10}$$

$$Q_{\mathrm{SC,max}} = \sigma w x_{\max} \tag{11}$$

$$V'_{\max} = \frac{\sigma x_{\max}}{\varepsilon_0 l} d_0 \tag{12}$$

SEC:

$$V_{\mathrm{OC,max}} = V'_{\max} = \sigma w l \frac{C_2(x_{\max})}{C_1(x_{\max})C_2(x_{\max}) + C_2(x_{\max})C_3(x_{\max}) + C_3(x_{\max})C_1(x_{\max})} \tag{13}$$

$$Q_{\mathrm{SC,max}} = \sigma w l \frac{C_2(x_{\max})}{C_1(x_{\max}) + C_2(x_{\max})} \tag{14}$$

CFT:

$$Q_{\mathrm{SC,max}} = \sigma w l \left[\left(\frac{1}{C_2(x_{\max})} + \frac{2}{C_3(x_{\max})} \right) C_{\mathrm{Total}}(x_{\max}) - \left(\frac{1}{C_2(0)} + \frac{2}{C_3(0)} \right) C_{\mathrm{Total}}(0) \right] \tag{15}$$

$$V_{\mathrm{OC,max}} = \frac{Q_{\mathrm{SC,max}}}{C_{\mathrm{Total}}(x_{\max})} \tag{16}$$

$$V'_{\max} = \frac{Q_{\mathrm{SC,max}}}{C_{\mathrm{Total}}(0)} \tag{17}$$

The capacitances listed above were calculated by considering 1-side (1S) and 2-side (2S) side effects of the non-ideal parallel capacitor. The equations are listed below[5]:

$$C_{\mathrm{1S}} = \varepsilon_0 \varepsilon_{\mathrm{r}} \left\{ \frac{lw}{d} + \frac{l}{\pi} \left[1 + \ln\left(1 + 2\pi \frac{w}{d} + \ln\left(1 + 2\pi \frac{w}{d} \right) \right) \right] \right\} \tag{18}$$

$$C_{2S} = \varepsilon_0\varepsilon_r \left\{ \frac{lw}{d} + \frac{l}{\pi}\left[1+\ln\left(1+2\pi\frac{w}{d}+\ln\left(1+2\pi\frac{w}{d}\right)\right)\right] + \frac{w}{\pi}\left[1+\ln\left(1+2\pi\frac{l}{d}+\ln\left(1+2\pi\frac{l}{d}\right)\right)\right]\right\} \quad (19)$$

Here, l, w and d represent length, width and gap distance of the parallel capacitor.

And then, the largest possible output energy per cycle E_m was calculated by Equation (2) in the paper and the structural FOM with respect to different x_{max} was calculated by Equation (5) in the paper.

Supplementary Note 6: The supplementary discussions about tested materials: For some materials such as polydimethylsiloxane (PDMS), there are always liquid gallium or galinstan droplets adhered to the material surface during contact-separation process; then the surface charge density of the material cannot be accurately tested using liquid gallium or galinstan, since the droplets may take part of triboelectric charges. Instead, other liquid metals, such as mercury, could be used for charge density measurement of these materials, as reported recently.[6] The same method is still valid for the measurement of PDMS charge density using mercury.

Supplementary Note 7: FEP as the most triboelectric negative material: FEP and polytetrafluoroethylene (PTFE) both have pure fully fluorinated carbon-fluorine structures, and they have nearly the same properties including triboelectric properties. In triboelectric series, they are usually listed as the same material with the commercial name of Teflon (such as: https://www.trifield.com/content/tribo-electric-series/). Here since FEP is softer and more transparent than PTFE, we believe FEP will be more commonly

used in future development of flexible transparent devices than PTFE. Therefore, tests based on FEP film should have equal significance.

Supplementary References:

1 Niu, S. *et al.* Theoretical Investigation and Structural Optimization of Single-Electrode Triboelectric Nanogenerators. *Advanced Functional Materials* **24**, 3332-3340, doi:10.1002/adfm.201303799 (2014).

2 Niu, S. *et al.* Theory of freestanding triboelectric-layer-based nanogenerators. *Nano Energy* **12**, 760-774, doi:10.1016/j.nanoen.2015.01.013 (2015).

3 Niu, S. *et al.* Theory of Sliding-Mode Triboelectric Nanogenerators. *Advanced Materials* **25**, 6184-6193, doi:10.1002/adma.201302808 (2013).

4 Niu, S. *et al.* Theoretical study of contact-mode triboelectric nanogenerators as an effective power source. *Energy & Environmental Science* **6**, 3576-3583, doi:10.1039/C3EE42571A (2013).

5 Li, Y., Li, Y. H., Li, Q. X. & Zi, Y. Y. Computation of electrostatic forces with edge effects for non-parallel comb-actuators. *J Tsinghua Univ (Sci & Tech)* **43**, 1024-1026, 1030 (2003).

6 Tang, W. *et al.* Liquid-Metal Electrode for High-Performance Triboelectric Nanogenerator at an Instantaneous Energy Conversion Efficiency of 70.6%. *Advanced Functional Materials* **25**, 3718-3725, doi:10.1002/adfm.201501331 (2015).
